# Quantification of Phytochemicals from Commercial* Spirulina* Products and Their Antioxidant Activities

**DOI:** 10.1155/2016/7631864

**Published:** 2016-01-04

**Authors:** Naif Abdullah Al-Dhabi, Mariadhas Valan Arasu

**Affiliations:** Department of Botany and Microbiology, Addiriyah Chair for Environmental Studies, College of Science, King Saud University, P.O. Box 2455, Riyadh 11451, Saudi Arabia

## Abstract

The present study aimed to profile the polyunsaturated fatty acids, sugars, free amino acids, and polyphenols in 37 varieties of* Spirulina *commonly available in the market using gas chromatography and high performance liquid chromatography. In addition, the biological potentials of the* Spirulina* samples were evaluated by analysing the* in vitro* antioxidant activities using various analytical techniques. The analyses revealed the presence of 13 polyunsaturated fatty acids, 18 amino acids, 7 sugars, and polyphenols. The polyunsaturated fatty acids contents were varied between* Spirulina* samples. The total polyunsaturated fatty acids amount was 4.25 mg/100 g, and the average among of sapienic acid detected was 2.25 mg/100 g, which was followed by linoleic acid (16.7%) and *γ*-linolenic acid (14%). Among the 7 sugars, the hexose levels were the highest (73.85%). The total amino acids contents ranged from 11.49 to 56.14 mg/100 g, and the individual essential amino acids accounted for 17% to 39.18%. The “natural” tablets exhibited the highest polyphenols levels (24 mg/g). All of the* Spirulina* samples expressed dose-dependent antioxidant activities. The polyunsaturated fatty acids, sugars, free amino acids, and polyphenols contents varied widely, and the variations in these compounds between the* Spirulina* samples were significant.

## 1. Introduction

Cyanobacteria also include unicellular organisms and all of them are not spiral shaped (*Spirulina* is spiral shaped). They grow naturally in the water of warm climates and are primarily cultivated in ponds and small lakes. In contrast to other living organisms, microalgae or* Spirulina* is one of the microalgae and is not the only microalga which do not require organic, inorganic, nutrient, and other carbon sources for growth and can survive in higher alkaline pHs and in greater bicarbonate and carbonate concentrations. Beginning in the sixteenth century, cyanobacteria have been used as a traditional food source for African and Mexican people. Among the microalgae,* Spirulina maxima* (*Arthrospira maxima*)*, Spirulina platensis* (*Arthrospira platensis*), and* Spirulina fusiformis* (*Arthrospira fusiformis*) are the most widely cultivated species around the world and are widely used as health foods, food additives, and potential sources of high value chemicals and pharmaceutical metabolites [1]. Each year, more than three thousand tons of* Spirulina* are cultivated around the world for human nutrition and the production of other fine commodity chemicals [2]. Several similar studies with market samples of* Spirulina* have been carried out earlier and data are available [3]. In recent years, people have been interested in consuming* Spirulina* in tablet and powder forms due to its relatively high contents of protein (58%), carbohydrates (30%), fat (8%), dietary fibres (3%), sugars (3%), vitamins (<1%), and phytochemicals [4, 5].* Spirulina *contains fatty acids such as linoleic acid, docosahexaenoic acid, eicosapentaenoic acid, arachidonic acid, and stearidonic acid, respectively.* Spirulina* also contains moderate amounts of vitamins such as vitamin A, vitamin C, vitamin E, vitamin B12, thiamine, nicotinamide, pyridoxine, riboflavin, and folic acid and beneficial pigments, such as chlorophyll-a, zeaxanthin, diatoxanthin, 3′-hydroxyechinenone, echinenone, beta-carotene, xanthophyll, canthaxanthin, c-phycocyanin, beta-cryptoxanthin, myxoxanthophyll, oscillaxanthin, phycobiliproteins, and allophycocyanin [5, 6]. However, the nutritional contents of* Spirulina* depend on the cultivation conditions and the processing methods. The nutritional components and other phytochemicals in* Spirulina* primarily exhibit anti-inflammatory, antioxidant, antidiabetic, neuroprotective, hepatoprotective, and anticancer activities [7]. The regular consumption of* Spirulina* ameliorates the symptoms of premenstrual cycles in women and the symptoms of amyotrophic lateral sclerosis.* Spirulina* prevents allergic reactions and aids in the removal of metals from the body. A recent study suggested that* Spirulina* helps to bind radioactive elements and is useful for protecting the human body from exposure to radiation therapy. The phenolic compounds present in the* Spirulina* are primarily involved in the redox mechanism and act as hydrogen donors, reducing agents, metal chelator singlets, and oxygen quenchers [8]. Therefore, phenolic compounds can prevent the formation of ROS and reactive nitrogen species, which include free radicals, such as hydroxyl and superoxide anions and nitric oxide, and nonfree radical species, such as hydrogen peroxide and nitrous acid. The development of phenolic compounds as antioxidants for the treatment of various human diseases has increased. Therefore, there is an urgent need to identify novel antioxidant molecules with fewer side effects and significant hepatoprotective effects [9]. To overcome disorders, the regular consumption of natural health-promoting foods, such as* Spirulina* tablets or powders, is advised.* In vitro* studies demonstrated that the* Spirulina* and* Nestoc* species have several therapeutic properties, due to their ability to scavenge superoxide and hydroxyl radicals and inhibit lipid peroxidation [10, 11]. Therefore, the present study aimed to investigate the metabolite profiles and antioxidant properties of 37 commercially available* Spirulina* samples.

## 2. Materials and Methods

### 2.1. Chemicals and Solvents

Standard methyl esters of fatty acids were obtained from Supelco (37 Component FAME Mix). Triglyceride (IS, C11:0 triundecanoin) was purchased from Nu-Chek Prep (Elysian, MN, USA). BF3-methanol (10%, w/w) was procured from Supelco (Bellefonte, PA, USA). Analytical grade diethyl ether (DE), pyrogallol, petroleum ether (PE), chloroform, and ethanol were purchased from Sigma-Aldrich Chemical Co. (St. Louis, Mo., U.S.A.). Thirty-seven* Spirulina* samples with different countries of origin in the forms of tablets and capsules were procured from specialist shops (Table 1). All the studied* Spirulina* samples were procured from different markets in the world. The details of the samples were mentioned in Table 1.

### 2.2. Extraction of Lipids from the* Spirulina* Samples

The total lipids in the* Spirulina* samples were extracted according to the following modified method of Mossoba et al. (2003) [12]. Briefly, one gram of finely powdered sample with 2 mL pyrogallol solution (in ethanol 95%, 50 mg/mL) and 1 mL triglyceride internal standard solution (IS: C11:0 triundecanoin 5 mg/mL in iso-octane) was transferred into a 50 mL tube. After proper mixing, 10.0 mL 8.3 M HCl was added into the tube, which was then incubated in a shaking water bath at 70–80°C for 2 h. During the incubation, the contents of the tubes were intermittently mixed to release the fat from the walls of the tubes. After incubation, the samples were allowed to cool at room temperature and mixed with 15 mL diethyl ether (DE). The DE layer was then separated and filtered in the column using Na_2_SO_4_ and petroleum ether (PE). Subsequently, the collected PE layers were slowly evaporated using a nitrogen stream and used for the extraction of the fatty acids.

### 2.3. Extraction and Quantification of the Fatty Acids

The total lipids in the* Spirulina* were extracted according the method of Mossoba et al. (2003) with modifications [12]. Briefly, the extracted lipids were saponified with 0.5 N NaOH in methanol (1.5 mL) for 5 min at 100°C and cooled at room temperature. After cooling, the samples were treated with 2 mL of BF3-methanol and incubated at 100°C for 10 min and allowed to cool at room temperature. The samples were then thoroughly vortexed with 2 mL of isooctane and 1 mL of saturated NaCl solution for 10 min. Next, the upper isooctane layer was carefully transferred into tubes and injected into a Hewlett-Packard 6890 series gas chromatograph (GC) equipped with an autoinjector and a flame-ionization detector (Agilent Technologies, Little Falls, Del., USA). The fatty acids were separated in a fused-silica capillary column (SP-2560, 100 m × 0.25 nm × 0.2 *μ*m film thickness, Supelco, USA). The GC oven was heated to 100°C and held for 4 min and then further increased to 240°C at a rate of 3°C/min and held at 240°C for 15 min. The injector and detector temperatures were set at 225°C and 285°C, respectively. The mobile gas (helium) applied at a flow rate of 0.75 mL/min. The concentrations of the individual fatty acids were calculated based on the relative retention times of the standard mixtures. The conversion of FAMEs to corresponding fatty acids are shown in Table 2.

The response factor (*R*_*i*_) of each fatty acid was calculated as follows:

(1) $$\begin{array}{c} R_{i}=\frac{{Ps}_{i}}{{Ps}_{C11:0}}\times \frac{W_{C11:0}}{W_{i}}, \end{array}$$

where *Ps*_*i*_ is peak area of individual fatty acid in mixed FAMEs standard solution; *Ps*_C11:0_ is peak area of C11:0 fatty acid in mixed FAMEs standard solution; *W*_C11:0_ is weight of internal standard in mixed FAMEs standard solution; and *W*_*i*_ is weight of individual FAME in mixed FAMEs standard solution.

The amounts of the individual compounds in the test samples were calculated as follows:

(2) $$\begin{array}{c} W_{FAMEi}=\frac{{Pt}_{i}\times {Wt}_{C11:0}\times 1.0067}{{Pt}_{C11:0}\times R_{i}}, \end{array}$$

where *Pt*_*i*_ is peak area of the fatty acid *i* in the test portion; *Wt*_C11:0_ is weight of C11:0 in the internal standard added to test portion, g; 1.0067 is conversion of the internal standard from triglyceride to FAME; and *Pt*_C11:0_ is peak area of C11:0 in the internal standards in the test portion.

The weight of the fatty acid (*W*_*i*_) was determined as follows:

(3) $$\begin{array}{c} W_{i}=W_{FAMEi}\times f_{FAi}, \end{array}$$

where *f*_FA*i*_ is conversion factors for the conversion of the FAMEs to their corresponding fatty acids.

### 2.4. Extraction and Quantification of Sugars Using HPLC

The carbohydrates present in the* Spirulina* samples were quantified according to the following the standard method [14]. Briefly, a 100 mg portion of the powdered sample was mixed with 10 mL sterile distilled water and boiled at 100°C for one hour. After heating, the debris was separated by centrifugation at 10,000 rpm for 10 min. Next, the debris-free solution was mixed with 10% 5% trichloroacetic acid (TCA) and incubated at room temperature for 10 min. Subsequently, the samples were centrifuged at 10,000 rpm for 10 min and then filtered through a 0.45 *μ*m PTFE syringe filter for HPLC analysis (HP1100 Agilent Co., USA). The filtered samples were separated through a 300 × 7.8 mm Aminex HPX-87H (Bio-Rad; Hercules, CA, USA) column at 60°C using a 5.0 mM H_2_SO_4_ monophasic solvent system with a flow rate of 0.5 mL/min and a column wavelength of 220 nm. The injection sling was 10 *μ*L. The quantifications of the individual sugars were based on the peak areas and calculated as equivalents of standard compounds.

### 2.5. Extraction and Quantification of the Free Amino Acids Using HPLC

Individual free amino acids were extracted and quantified according to the method of Park et al. (2014) with modifications [15]. Briefly, a 100 mg portion of a fine powdered sample was mixed with 1.2 mL of 5% trichloroacetic acid (TCA) in a 2 mL Eppendorf tube and vigorously shaken for 5 min. The slurry sample was incubated at room temperature for 60 min, and the upper layer was then separated by centrifugation. The collected samples were diluted with 0.1 M HCl and then filtered through a 0.45 *μ*m PTFE syringe filter. The filtrate was then analysed by HPLC (Agilent Technologies, Palo Alto, CA). The HPLC analyses of free amino acids were conducted according to the “rapid, accurate, sensitive, and reproducible HPLC analysis of amino acids analysis” method with Zorbax Eclipse-AAA columns using an Agilent 1100 HPLC system. Briefly, the separation of the free amino acids was performed on a Zorbax Eclipse AAA analytical column. The oven temperature of the column was set at 40°C, and the detection wavelength was set a 338 nm. The injection volume was 10 *μ*L. The mobile phase consisted of a mixture of 40 mM NaH_2_PO_4_ (pH 7.8, solvent A), and solvent B (ACN, MeOH, and water at a 45 : 45 : 10 v/v/v ratio) was passed at a rate of 2.0 mL/min. The HPLC separation parameters were as follows: 0 min, 0% B; 0–1.9 min, 0% B; 1.9–21.1 min, 57% B; 21.1–21.6 min, 100% B; 21.6–25 min, 100% B; 25–25.1 min, 0% B; and 25.1–30 min, 0% B. A sample with an amino acid content of 50 pmoL/*μ*L was used as the standard. The quantifications of the different amino acids were based on the peak areas and were calculated as equivalents of the standard compounds. All contents are expressed as milligrams *∗* gram/fresh weight (FW).

### 2.6. Total Polyphenol Analysis

The total polyphenols were estimated according to the method of Folin-Ciocalteu (modified from Lin, and Tang, 2006) [16]. Briefly, 10 mg of the sample was dissolved in 1 mL of methanol with 2 mL of Folin-Denis reagent and 35% sodium carbonate (Na_2_CO_3_). The mixture was stored at room temperature for 30 min. The absorbance was measured with an UV-Vis spectrophotometer at 750 nm. The total polyphenols were calculated as gallic acid equivalents based on a calibration curve for gallic acid (0, 25, 50, and 100 *μ*g/mL) using the following equation that was based on the calibration curve: *y* = −0.9706*x* + 3.8935 (*R* ^2^ = 0.9992).

### 2.7. *In Vitro* Antioxidant Assays

#### 2.7.1. Preparation of the* Spirulina*

One gram of the fine powder sample was mixed with 5 mL of ethanol in a screw-cap tube by vortexing for 5 min and then kept in an orbital shaker at 150 rpm for 24 h at room temperature for thorough extraction. After incubation, the samples were centrifuged at 13,000 rpm for 15 min at 4°C. The resulting supernatant was vacuum evaporated at 30°C, and the resulting extract was used for the antioxidant assays.

#### 2.7.2. Reducing Power Activity Assay

The reducing power assay was performed according to the method of Oyaizu (1986) [17]. Volume of 100 *µ*L of various concentrations (20–100 *μ*g/mL) of the samples was mixed with phosphate buffer (2.5 mL) and 1% potassium ferricyanide (2.5 mL) and incubated at 50°C for 20 minutes. After incubation, 2.5 mL of 10% trichloroacetic acid was added, and the samples were centrifuged at 3000 rpm for 10 min. The upper layer of the solution (2.5 mL) was mixed with distilled water (2.5 mL) and a freshly prepared 0.1% ferric chloride solution (0.5 mL) and measured at an absorbance at 700 nm. The control was prepared in a similar manner, but the sample was excluded. Vitamin C at various concentrations was used as a standard. Increases in the absorbance of the reaction mixture indicated increases in reducing power.

#### 2.7.3. DPPH Radical Scavenging Activity Assay

The DPPH radical scavenging assay performed according to the method of Hatano et al. (1988) [18]. Briefly, 100 *µ*L of the sample and vitamin C (concentration 100–500 *µ*g/mL) was mixed with 200 *µ*L of freshly prepared DPPH solution (1 mg/mL in methanol) and incubated at room temperature in the dark for 30 minutes. The controls included only deionized water and the DPPH solution. The absorbances of the resulting solutions were measured in triplicate at 517 nm following centrifugation at 12000 rpm for 10 min.

The scavenging activity was calculated as follows:

(4) $$\begin{array}{c} scavenging\;\;activity\left(\;\%\;\right)=\left[\;1-\frac{\left(A_{0}-A_{1}\right)}{A_{2}}\;\right]\times 100, \end{array}$$

where *A*_0_ is absorbance of sample, *A*_1_ is absorbance of blank, and *A*_2_ is absorbance of control.

#### 2.7.4. Hydroxyl Radical-Scavenging Activity Assay

The hydroxyl radical-scavenging assay was performed according to the method of Elizabeth and Rao (1990) with slight modification [19]. The reagents for the assay were freshly prepared. Briefly, one millilitre samples of the working solutions that consisted of different ratios of the extract were mixed with 100 mL of 28 mM 2-deoxy-2-ribose in phosphate buffer (pH 7.4), EDTA (1.04 mM, 1 : 1, v/v), 100 mL H_2_O_2_ (1 mM), 200 mL of FeCl_3_ (200 mM), and 100 mL ascorbic acid (1 mM). The resulting solutions were mixed evenly, and the reaction mixtures were incubated at 37°C for 1 h. The degradation of deoxyribose was determined by reading the absorbance at 532 nm against the blank solution using a microplate reader (BioRad). Vitamin C was used as a positive control. The experiments were conducted in triplicate. The scavenging activities were calculated according to (4). 

## 3. Results and Discussion

### 3.1. Variations in the Fatty Acid Contents

**Figure 1:**
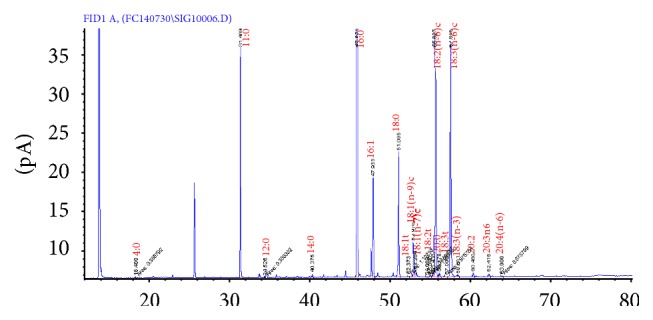
Gas chromatograms of the fatty acids identified in the* Spirulina* samples. The peaks numbers refer to the individual fatty acids listed in Table 2.

The total lipid contents were extracted from the* Spirulina* samples, and the individual fatty acid compositions of the samples were analysed with gas chromatography. A gas chromatograph coupled with a flame ionization detector guided the identification of the following 10 unsaturated and fatty acids (sapienic acid, palmitoleic acid, elaidic acid, oleic acid, vaccenic acid, linolelaidic acid, linoleic acid, eicosenoic acid, *γ*-linolenic acid, and dihomo-gamma-linolenic acid) and three saturated and fatty acids (myristic acid, stearic acid, and eicosadienoic acid) (Figure 1 and Table 2). The individual PUFA contents were quantified by comparing the standard fatty acids with their indices. The PUFA contents in the* Spirulina* samples ranged from 3.01 (DXN Marketing, capsules) to 7.41 g/100 g (21st Century HealthCare, Inc., Arizona, tablets; Table 4). Interestingly, there were comparatively lower amounts of *γ*-linolenic acid in the studied* Spirulina* samples; this acid accounted for an average of 14% of the total PUFAs. The amounts of *γ*-linolenic acid ranged from 0.16 g/100 g (General Nutrition Corp., Pittsburgh, capsules) to 1.24 g/100 g (21st Century HealthCare, Inc., Arizona, tablets). However, Mühling et al. (2005) reported palmitic acid (C16:0) noted as the dominant fatty acid in wild* Spirulina* samples [20]. In our study sapienic acid been noted as the major fatty acids. Many* in vitro* studies have confirmed that *γ*-linolenic acid can be used to effectively lower cholesterol and treat atopic eczema, breast cancer, and premenstrual disorder [21–25]. Recently, Sajilata et al. (2008) extracted and purified S. platensis active components via lipid fractionation, silica gel column purification, and thin-layer chromatographic methods [26]. It has been reported that *α*-linolenic acid and *γ*-linolenic acid are required for the survival of animals and humans, Patil et al. (2007) [27]. Patil et al. (2007) profiled the individual PUFAs from* Bacillariophyceae, Cyanophyceae, Rhodophyceae, Xanthophyceae, Cryptophyceae, Prymnesiophyceae, Eustigmatophyceae, and Chlorophyceae* microalgae and suggested that the cultivation conditions, particularly light intensity, and other nutritional components exert important effects on the PUFA compositions [27]. Many companies process microalgae and supply the results in the forms of capsules and tables to the market. Recently, the interest in the use of* Spirulina* tablets as energy foods has been renewed due to the relatively high contents of protein, phytochemicals, and other nutrients in such tablets. Efforts should be made to analyse the metabolite profiles of the commercially available* Spirulina* products because, in a previous study, we confirmed that trace amounts of heavy metals that could cause serious health problems for consumers are present in some commercially available* Spirulina* samples (Table 3) [13].

### 3.2. Quantification of Individual Sugars by HPLC

Hexose (i.e., glucose, fructose, galactose, and rhamnose), pentose (i.e., xylose and ribose), and disaccharide sugars were extracted from the 37* Spirulina* samples and clearly baseline eluted by HPLC. The quantitative results revealed that glucose, fructose, and sucrose were present in the greatest amounts followed by xylose, ribose, galactose, and rhamnose. The total sugar contents of the* Spirulina* samples ranged from 309 to 1221.67 mg/100 g (Table 4). Together, glucose, fructose, galactose, and rhamnose accounted for an average of 73.85% of the total sugar contents. Among the major individual sugars, glucose accounted for an average of 351 mg/100 g and 52% of the total sugar contents. Similarly, Chaiklahan et al. (2013) reported that rhamnose and glucose account for 53% and 13% of the total sugars, respectively [28]. The rhamnose contents varied from 8 to 58 mg/100 g of the total sugars, accounting for an average of 6.5% [29]. The results indicated that, among the pentose sugars, xylose (average 9.08%) and ribose (average 4.75%) were the major components in the* Spirulina* samples. The final outcome of this study is that the variations in the individual sugar contents between the* Spirulina* samples were acceptable due to the processing conditions of the each commercial* Spirulina *product. Moreover, a literature stated that the extraction of total polysaccharides and other monosaccharides from* Spirulina* followed by the quantification of the individual sugar molecules identified rhamnose as the predominant sugar followed by glucose and fructose [29].

### 3.3. Quantifications of the Individual Amino Acids by HPLC

**Figure 2:**
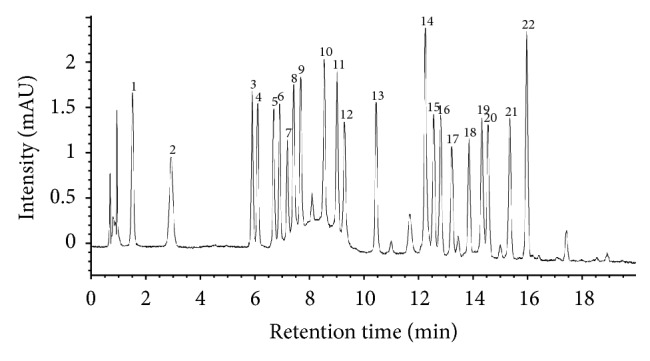
HPLC chromatogram of the standard free amino acids. The peaks numbers refer to the free amino acids listed in Table 5. Peaks numbers 1, aspartate; 2, glutamate; 3, asparagine; 4, serine; 5, S-methylmethionine (vitamin U); 6, glutamine; 7, histidine; 8, glycine; 9, threonine; 10, arginine; 11, alanine; 12, gamma-aminobutyric acid (GABA); 13, tyrosine; 14, cystine; 15, valine; 16, methionine; 17, norvaline; 18, tryptophan; 19, phenylalanine; 20, isoleucine; 21, leucine; 22, lysine.

HPLC analyses were used to quantify 22 free amino acids, including aspartate, asparagine, serine, glutamine, histidine, glycine, threonine, arginine, alanine, *γ*-aminobutyric acid (GABA), tyrosine, valine, cystine, methionine, tryptophan, phenylalanine, isoleucine, leucine, and lysine, in the* Spirulina* samples, but the separation profiles revealed that only 18 free amino acids were detectable in the samples, but the other free amino acids did not detected which may be not present in the samples (Table 5 and Figure 2). Since the samples were marketed by the trademark of different companies and the nutrient profile of each sample would be varied, these results revealed that the amounts of total free amino acids in the 37* Spirulina* samples ranged from 11.49 mg/100 g to 56.14 mg/100 g. The essential amino acid content averages ranged from 2.06 to 31.72 mg/100 g and contributed averages that ranged from 17.0 to 39.18% of the total amino acids. Among the essential amino acids, leucine was identified as predominant (0.53 to 7.59 mg/100 g) and accounted for more than 30% of the essential amino acids. The* Spirulina* tablets marketed in India under the brand name “Dharain Pharmaceuticals” exhibited the greatest essential amino acid contents. However, the brand name products exhibited values that were comparatively lower than the maximum observed values. Vitamin U, methionine, norvaline, and tryptophan were not observed in the samples. The essential amino acid compositions of the microalgae were very similar to the reported protein contents [30]. Clément et al. (1967) determined the individual and total amino acid contents in* S. maxima* [31]. This study reported that aspartate was dominant in the* Spirulina *samples and that histidine, cystine, tryptophan, and methionine were observed at the lowest levels. This report found a level of aspartate that was similar to that of another report. In general, many companies market* Spirulina* samples as nutraceutical food; however, there is an urgent need to know the nutritional compositions of each of these* Spirulina* products [32]. This study confirmed that the amino acid compositions of* Spirulina* samples varied with the companies that produced them. Therefore, amino acid-rich samples should be consumed by humans to maintain their health.

### 3.4. Determination of the Total Phenolic Compounds

**Figure 3:**
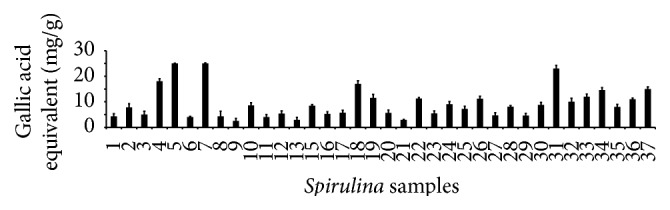
Determination of the total phenolic compounds of the 37 varieties (Table 1) of* Spirulina* (*n* = 3).

Figure 3 shows the total phenolic compounds calculated as equivalents to gallic acid. The results revealed that the distributions of the total phenolic compounds varied between the commercial products. The products ranged from 2.4 mg/g (21st Century HealthCare, Inc., Arizona) to 24.4 mg/g (source Naturals, Inc., Santa Cruz, California). The highest level of total polyphenol contents (24 mg/g) was observed in the tablets procured from source Naturals, Inc., Santa Cruz, California. Miranda et al. (1998) claimed that the main phenolic compounds, namely, chlorogenic acid, synaptic acid, salicylic acid, trans-cinnamic acid, and caffeic acid were commonly present in* Spirulina* [33]. The present study also coincides with the report of Miranda et al. (1998) [33]. However, the metabolic pathways for the formation of phenolics compounds in* Spirulina* and their importance are still unknown [34]. The polyphenols contained the ideal chemical structures and different bioactivities that included anti-inflammatory, antiviral, antioxidant, antithrombotic, vasodilatory, and anticarcinogenic properties [35]. Wu et al. (2005) demonstrated that the presence of total phenolic components and other metabolites are related to antioxidant properties [36].

### 3.5. Antioxidant Properties

**Figure 4:**
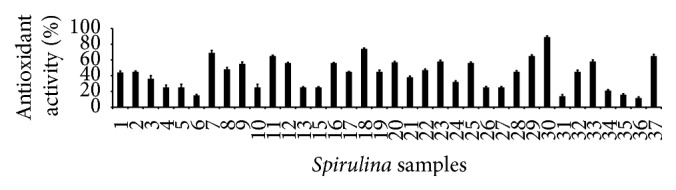
Antioxidant activities of the 37 varieties (Table 1) of* Spirulina* by DPPH radical scavenging activity assay (*n* = 3).

**Figure 5 fig5:**
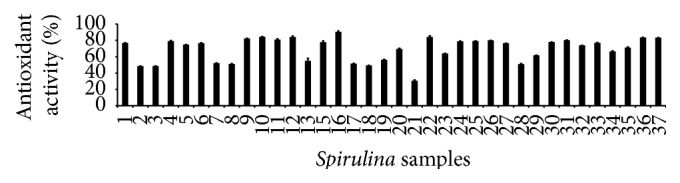
Antioxidant activities of the 37 varieties (Table 1) of* Spirulina* by reducing power activity assay (*n* = 3).

**Figure 6:**
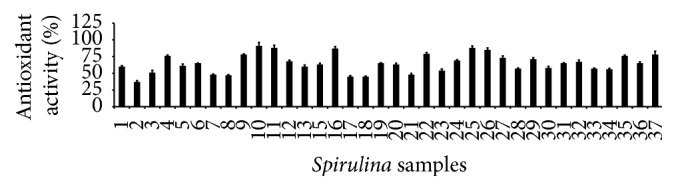
Antioxidant activities of the 37 varieties (Table 1) of* Spirulina* by* hydroxyl *radical-scavenging activity assay (*n* = 3).

The results revealed that the antioxidant properties of the* Spirulina* samples were dose-dependent (Figures 4 [Fig fig5]–6). The DPPH assay and hydroxyl scavenging assay results revealed that all the* Spirulina* extracts showed the activity in a concentration-dependent manner. Numbers of antioxidant metabolites are present in plants and* Spirulina*. The scavenging abilities and reductive properties of the* Spirulina* samples exhibited dose-dependent activities. Metabolites especially those having the phenolic functional group in their chemical structure have been reported to show many useful properties, including anti-inflammatory activity, oestrogenic activity, enzyme inhibition, antiallergic activity, antioxidant activity, vascular activity, and cytotoxic antitumour activity. The results from three antioxidant assays of the 37 samples were not correlated with the total phenolic compounds or the other determined compounds. The results indicated that the antioxidant potentials were not significantly correlated with their total phenolic compounds (data were not shown), because the antioxidant activity of the samples was not directly proportional with respect to the total phenolic compounds results. The regular consumption of antioxidant-containing food additives helps to slow oxidative stress and minimize the spread of oxidative stress-related diseases [37]. The antioxidant compounds, such as phycobilins and phycocyanins, that are present in* Spirulina* exert their actions by scavenging free radicals by acting as hydrogen, peroxyl radical, and peroxynitrite acceptors. These antioxidant compounds also inhibit the activities of catalytic enzymes, such as lipoxygenase and cyclooxygenase, or enhance the activity of enzymes, such as glutathione peroxidase, catalase, and superoxide dismutase [38]. Wu et al. (2005) reported that* Spirulina* extracts exhibited greater antioxidant properties due to the presence of various phenolic compounds [36]. A number of cyanobacteria especially the species of* Chlorella* are believed to be useful as excellent food sources with antioxidant activities by modern researchers [39]. Due to its rich vitamin, protein, phenolic compound, polyunsaturated fatty acid, and other microelement contents,* Spirulina* could be used as a better nutrient food by consumers.

## 4. Conclusions

Thirteen unsaturated fatty acids, 19 free amino acids, 7 sugars, and the total polyphenolic components were separated and identified from 37* Spirulina* samples using GC and HPLC methods. The contents of each metabolite were quantified, and remarkable variations in the individual metabolites were observed between the different varieties. Specifically the* Spirulina* tablets distributed by 21st Century HealthCare, Inc., were relatively suitable due to their abundance of fatty acids, sugars, amino acids, and polyphenols. The* in vitro* antioxidant activity results confirmed that the activities were dose-dependent. The* Spirulina* products that are available on the market are rich in antioxidant polyphenolic components and are suitable choices for regular consumption. The presence of individual phenolic compounds in the different products should be studied because these metabolites are used for the treatment of stress-related diseases and cardiovascular disorders.

## Figures and Tables

**Table 1:** *Spirulina* products lists and their country of origin.

S. number	Product type	Manufacturing company	Country of origin	Web address
1	Tablets	TAAU Australia Pvt Ltd., NT	Australia	http://www.australianspirulina.com.au/
2	Capsules	General Nutrition Corp., Pittsburgh	USA	http://www.gnc.com/
3	Capsules	Nature's Way Products, Inc., Springville, Utah	USA	http://www.naturesway.com
4	Tablets	Good ‘N Natural, New York	USA	http://www.goodandnaturalstore.com
5	Tablets	Now Foods, Bloomingdale	USA	http://www.nowfoods.com
6	Tablets	Nature Pure, Inc., Larkspur, California	USA	
7	Tablets	Source Naturals, Inc., Santa Cruz, California	USA	http://www.sourcenaturals.com
8	Tablets	Jarrow Formulas, Los Angeles, CA	USA	http://www.jarrow.com
9	Tablets	Earthrise Nutritionals LLC, Irvine, CA	USA	http://earthrise.com/
10	Tablets	Nutrex Hawaii Inc., Kailua-Kona, Hawaii	USA	http://www.nutrex-hawaii.com
11	Capsules	Pure Planet Products, Inc., Long Beach, CA	USA	https://www.pureplanet.com
12	Tablets	Puritan's Pride, Inc., Oakdale, New York	USA	http://www.puritan.com
13	Capsules	21st Century HealthCare, Inc., Arizona	USA	http://www.21stcenturyvitamins.com
14	Tablets	Japan Algae Co., Ltd., Tokyo	Japan	http://www.sp100.com/
15	Tablets	All Seasons Health, Hampshire	United Kingdom	http://www.carehome.co.uk/
16	Capsules	Fushi Wellbeing Ltd., London	United Kingdom	http://www.fushi.co.uk
17	Tablets	Biovea, London	United Kingdom	http://www.biovea.com
18	Capsules	Parry Nutraceuticals, Chennai	India	http://www.parrynutraceuticals.com
19	Tablets	Lifestream International Ltd., Northcote, Auckland	New Zealand	http://www.lifestream.co.nz
20	Tablets	Green Health, Auckland	New Zealand	http://www.greenhealth.co.nz
21	Tablets	RBC Life Sciences, Inc., Burnaby, British Columbia (BC)	Canada	http://www.rbclifesciences.com
22	Tablets	Swiss Herbal Remedies Ltd., Richmond Hill, Ontario	Canada	http://www.swissnatural.com
23	Capsules	Herbal Select, Guelph, Ontario	Canada	http://www.herbalselect.ca
24	Capsules	Gourmet Nutrition F.B. Inc., STE-Julie (Quebec)	Canada	http://www.gourmetfb.com
25	Capsules	Terra Vita Fine Whole Herbs, Brampton, Ontario	Canada	http://dsld.nlm.nih.gov/
26	Capsules	DXN marketing	Malaysia	http://www.dxnmalaysia.com
27	Capsules	Hydrolina Biotech Pvt., India	India	http://www.hydrolinabiotech.com
28	Capsules	Prime Health Laboratories Ltd.	Australia	Not available
29	Tablets	Laurel Herbal products	India	Not available
30	Tablets	IMO Control Pvt Ltd.	India	http://www.imocontrol.in
31	Capsules	Acumen Pharmaceuticals Pvt	India	http://www.acumenpharm.com
32	Capsules	Bio-Life Organic *Spirulina*	Malaysia	https://healthreview2u.wordpress.com/2010/09/15/biolifespirulina/
33	Tablets	Dharain Pharmaceuticals	India	http://www.mihnati.com/
34	Tablets	21st Century HealthCare, Inc., Arizona	USA	http://www.21stcenturyvitamins.com/
35	Tablets	Zuellig Bharma SDW. BHD	Malaysia	http://www.zuelligpharma.com
36	Tablets	Kordels *Spirulina*	India	http://www.rakuten.com
37	Tablets	Elken Chewable	Malaysia	https://elken4mrt.wordpress.com

**Table 2:** Fatty acids contents (mg/100 g in different *Spirulina*).

Sample	FA													Total
	14:00	16:00	16:1	18:0	18:1t	18:1(n-9)c	18:1(n-7)c	18:2t	18:2(n-6)c	20:00	18:3(n-6)	20:02	20:3n6	
	*f*_FA*i*_													
	0.9421	0.9481	0.9477	0.9530	0.9527	0.9524	0.95201	0.9517	0.9524	0.9570	0.952	0.9565	0.9557	
1	0.01	1.88	0.22	0.29	0.00	0.09	0.02	0.01	0.65	0.01	0.67	0.01	0.01	2.5
2	0.00	2.07	0.10	0.09	0.00	0.09	0.02	0.01	0.32	0.01	0.16	0.01	0.01	2.36
3	0.01	1.97	0.11	0.34	0.01	0.11	0.05	0.04	0.75	0.01	0.67	0.00	0.00	2.55
4	0.01	2.55	0.27	0.06	0.00	0.05	0.01	0.01	0.85	0.00	0.73	0.01	0.01	2.94
5	0.01	1.89	0.17	0.30	0.01	0.07	0.02	0.01	0.62	0.00	0.53	0.00	0.01	2.44
6	0.01	1.90	0.31	0.13	0.01	0.09	0.04	0.01	0.73	0.01	0.70	0.01	0.02	2.45
7	0.01	2.38	0.17	0.79	0.00	0.06	0.02	0.01	0.55	0.01	0.46	0.01	0.01	3.42
8	0.02	2.81	0.14	0.85	0.00	0.15	0.02	0.01	0.80	0.01	0.50	0.01	0.01	3.98
9	0.01	2.00	0.18	0.11	0.00	0.14	0.07	0.01	0.65	0.01	0.40	0.01	0.01	2.45
10	0.01	2.80	0.14	0.45	0.00	0.09	0.01	0.01	0.87	0.01	0.73	0.01	0.01	3.5
11	0.01	2.10	0.23	0.14	0.00	0.16	0.06	0.01	0.60	0.01	0.37	0.01	0.01	2.65
12	0.01	2.12	0.25	0.06	0.00	0.14	0.07	0.01	0.78	0.01	0.52	0.01	0.01	2.59
13	0.01	2.36	0.27	0.40	0.00	0.11	0.02	0.01	0.80	0.01	0.82	0.01	0.01	3.16
14	0.01	2.48	0.24	0.05	0.00	0.13	0.02	0.02	0.99	0.01	0.82	0.01	0.01	2.92
15	0.01	1.78	0.28	0.04	0.00	0.14	0.01	0.01	0.62	0.01	0.58	0.00	0.00	2.26
16	0.01	2.95	0.15	2.13	0.01	0.11	0.03	0.02	0.67	0.02	0.55	0.00	0.00	5.37
17	0.02	1.91	0.10	0.06	0.01	0.15	0.03	0.03	0.70	0.01	0.57	0.00	0.01	2.25
18	0.02	1.95	0.15	0.17	0.00	0.08	0.03	0.03	0.64	0.01	0.60	0.00	0.00	2.38
19	0.02	2.04	0.12	0.16	0.01	0.14	0.03	0.03	0.68	0.01	0.51	0.00	0.01	2.49
20	0.02	2.12	0.12	0.25	0.01	0.12	0.04	0.04	0.71	0.01	0.66	0.00	0.01	2.64
21	0.01	1.81	0.26	0.21	0.01	0.05	0.02	0.01	0.65	0.01	0.62	0.01	0.01	2.35
22	0.00	1.43	0.22	0.04	0.00	0.12	0.02	0.01	0.54	0.00	0.53	0.01	0.01	1.81
23	0.01	3.27	0.14	1.11	0.00	0.18	0.07	0.01	0.76	0.02	0.44	0.01	0.01	4.73
24	0.01	2.58	0.28	0.06	0.00	0.07	0.02	0.02	0.74	0.01	0.59	0.01	0.01	3.01
25	0.01	2.49	0.26	0.06	0.00	0.10	0.03	0.01	0.72	0.01	0.58	0.01	0.01	2.93
26	0.01	1.73	0.23	0.05	0.00	0.10	0.03	0.01	0.44	0.00	0.39	0.00	0.01	2.12
27	0.02	2.17	0.25	0.06	0.01	0.12	0.06	0.01	0.75	0.01	0.60	0.01	0.01	2.63
28	0.01	2.98	0.23	1.35	0.00	0.13	0.03	0.02	0.90	0.02	0.52	0.01	0.01	4.72
29	0.01	1.58	0.11	0.07	0.00	0.17	0.04	0.01	0.53	0.01	0.49	0.01	0.01	1.95
30	0.02	2.36	0.14	0.05	0.01	0.17	0.03	0.03	0.83	0.01	0.65	0.00	0.00	2.75
31	0.01	1.88	0.15	0.05	0.00	0.19	0.03	0.01	0.54	0.00	0.40	0.01	0.01	2.28
32	0.01	1.75	0.19	0.07	0.00	0.08	0.01	0.01	0.59	0.00	0.49	0.01	0.01	2.1
33	0.01	2.29	0.33	0.06	0.00	0.10	0.03	0.01	0.78	0.01	0.70	0.01	0.01	2.8
34	0.04	3.95	0.21	0.15	0.02	0.23	0.08	0.07	1.37	0.01	1.24	0.01	0.01	4.59
35	0.02	2.10	0.31	0.08	0.00	0.16	0.12	0.01	0.55	0.01	0.60	0.02	0.01	2.68
36	0.01	2.62	0.15	0.08	0.00	0.18	0.04	0.03	1.04	0.01	0.58	0.01	0.01	3.05
37	0.02	2.14	0.14	0.05	0.01	0.11	0.04	0.04	0.77	0.00	0.77	0.00	0.01	2.46

Numbers 1 to 37 were the sample names (Table 1). Total fatty acids were considered only for PUFA. 14:00: myristic acid, 16:00: sapienic acid, 16:1: palmitoleic acid, 18:0: stearic acid, 18:1t: elaidic acid, 18:1(n-9)c: oleic acid, 18:1(n-7)c: vaccenic acid, 18:2t: linolelaidic acid, 18:2(n-6)c: linoleic acid, 20:00: eicosenoic acid, 18:3(n-6): *γ*-linolenic acid, 20:02: eicosadienoic acid, and 20:3n6: dihomo-gamma-linolenic acid.

**Table 3:** Content of heavy metals in the *Spirulina* samples available in the market.

Sample name	Amount mg/Kg dry weight						
	Nickel	Zinc	Mercury	Platinum	Magnesium	Manganese	Total
1	0.211	0.533	0.002	0.001	0.002	0.076	0.825
2	4.672	5.627	0.028	0.01	0.03	0.587	10.954
3	2.016	2.397	0.017	0.008	0.018	0.603	5.059
4	2.147	1.628	0.02	0.011	0.026	0.436	4.268
5	2.199	1.235	0.017	0.008	0.014	1.17	4.643
6	3.726	6.225	0.022	0.009	0.028	0.007	10.017
7	2.601	2.817	0.018	0.011	0.028	0.007	5.482
8	3.577	1.871	0.023	0.012	0.031	0.309	5.823
9	3.519	3.267	0.021	0.011	0.03	0.008	6.856
10	2.442	2.041	0.017	0.008	0.021	0.137	4.666
11	3.785	3.007	0.019	0.008	0.028	0.008	6.855
12	3.597	2.859	0.019	0.008	0.024	0.006	6.513
13	2.857	2.901	0.019	0.008	0.026	1.643	7.454
14	2.852	2.114	0.026	0.008	0.042	0.011	5.053
15	2.437	1.568	0.02	0.008	0.028	0.008	4.069
16	2.712	2.434	0.019	0.009	0.033	1.777	6.984
17	2.948	2.513	0.021	0.009	0.028	1.8	7.319
18	2.633	1.876	0.016	0.009	0.023	1.328	5.885
19	3.731	3.184	0.017	0.009	0.036	2.248	9.225
20	2.225	1.548	0.008	0.009	0.019	1.132	4.941
21	1.618	1.478	0.014	0.006	0.02	0.005	3.141
22	1.589	4.626	0.016	0.007	0.023	0.009	6.27
23	3.272	4.428	0.02	0.008	0.018	0.008	7.754
24	3.558	3.733	0.017	0.009	0.034	1.433	8.784
25	2.319	2.586	0.017	0.008	0.024	0.006	4.96

Numbers 1 to 37 were the sample names (Table 1). Samples 26–37 were not analyzed [13].

**Table 4:** Individual sugar contents (mg/100 g) in different *Spirulina*.

Sample	Sugar contents (mg/100 g)							
	Glucose	Fructose	Xylose	Galactose	Ribose	Sucrose	Rhamnose	Total
1	172 ± 14	38 ± 3.45	25 ± 1.50	15.5 ± 0.56	18 ± 1.25	54 ± 5.6	25 ± 0.0	342.5 ± 4.33
2	71 ± 13.89	44 ± 1.5	88 ± 4.22	78 ± 3.12	5 ± 0.00	65 ± 0.24	18 ± 0.23	363 ± 5.19
3	229.33 ± 27.59	59 ± 5.05	55 ± 1.60	7 ± 0.71	45 ± 3.00	54 ± 3.45	35 ± 0.89	466.11 ± 15.77
4	51.33 ± 4.16	68 ± 5.01	21 ± 1.11	54 ± 2.01	7 ± 0.03	29 ± 2.03	19 ± 1.1	249.77 ± 0.38
5	347.33 ± 6.43	15 ± 1.24	46 ± 3.4	8 ± 0.23	25 ± 0.25	84 ± 00	58 ± 3.2	581.11 ± 1.92
6	26 ± 3.46	26 ± 2.8	32 ± 1	9 ± 0.17	16 ± 0.00	72 ± 1.30	45 ± 1	222 ± 3.46
7	148.33 ± 10.41	34 ± 1.78	37 ± 4.03	45 ± 3.12	18 ± 1.87	80 ± 9.15	25 ± 0.28	366.44 ± 18.08
8	156.33 ± 16.29	15 ± 12.1	39 ± 5.45	11 ± 1.02	31 ± 1.64	45 ± 7.45	14 ± 0.02	333.11 ± 18.86
9	253 ± 5.20	29 ± 5.98	65 ± 8.4	24 ± 2.0	22 ± 0.03	62 ± 6.05	35 ± 0.96	463.33 ± 23.09
10	255.33 ± 10.02	78 ± 7.4	98 ± 1.2	NA	NA	34 ± 5.10	NA	528.44 ± 14.62
11	128.33 ± 2.89	51 ± 0.89	45 ± 7.1	23 ± 1.85	24 ± 0.07	86 ± 6.88	18 ± 1.12	377.11 ± 1.54
12	361.66 ± 6.6	49 ± 1.04	25 ± 0.90	14 ± 2.2	16 ± 0.08	95 ± .00	8 ± 0.32	589.55 ± 18.0
13	482.33 ± 5.8	35 ± 2.08	45 ± 5.03	18 ± 1.03	54 ± 1.09	NA	11 ± 0.78	730.77 ± 1.34
14	592.33 ± 6.15	26 ± 1.0	85 ± 5.23	44 ± 2.9	22 ± 1.07	29 ± 2.15	16 ± 0.47	811.44 ± 2.49
15	248.66 ± 8	59 ± 6.8	48 ± 4.9	11 ± 2.2	15 ± 0.3	67 ± 1.68	24 ± 4.9	470.89 ± 1.54
16	210.33 ± 9.5	72 ± 5.6	95 ± 0.12	10 ± 0.62	24 ± 1.3	49 ± 9.0	17 ± 0.63	472.44 ± 4.23
17	517 ± 24	93 ± 4.05	25 ± .52	12 ± 0.62	26 ± 4.2	52 ± 0.51	28 ± 1.03	754.33 ± 1.15
18	884.66 ± 26.5	15 ± 0.25	22 ± 0.7	18 ± 2.15	28 ± 4.12	58 ± 1.52	38 ± 1.08	1059.89 ± 3.27
19	338.33 ± 12.5	44 ± 1.14	14 ± 0.9	27 ± 3.12	11 ± 0.00	49 ± 3.02	26 ± 0.0	509.77 ± 0.38
20	752 ± 5.26	51 ± 4.6	16 ± 0.12	9 ± 0.25	22 ± 0.8	67 ± 3.07	45 ± .28	987.33 ± 21.93
21	968.66 ± 27.3	50 ± 2.54	18 ± 0.59	58 ± 8.12	24 ± 0.14	84 ± 10.1	19 ± 5.1	1207.22 ± 12.52
22	260 ± 22.9	43 ± 6.3	94 ± 1.26	65 ± 4.74	26 ± 2.95	55 ± 0.78	54 ± 2.90	599 ± 1.73
23	107.33 ± 7.09	24 ± 4.0	25 ± 0.45	22 ± 0.12	25 ± 0.05	78 ± 1.84	28 ± 1.05	303.11 ± 5.38
24	151.66 ± 7.6	52 ± 2.65	63 ± 0.40	45 ± 2.94	11 ± 0.32	37 ± 0.00	47 ± 0.01	402.22 ± 3.85
25	102.66 ± 16	68 ± 4.3	24 ± 1.45	48 ± 2	7 ± 0.05	81 ± 0.28	35 ± 0.25	361.89 ± 3.27
26	79.33 ± 11.15	94 ± 5.0	25 ± 2.09	56 ± 6.23	45 ± 1.10	55 ± 6.12	45 ± 1.7	399.77 ± 0.38
27	604.66 ± 5.3	12 ± 1.37	26 ± 4.23	44 ± 2.56	24 ± 0.00	84 ± 0.71	37 ± 0.32	830.55 ± 0.96
28	901.66 ± 7.6	15 ± 0.7	45 ± .23	25 ± 0.45	10 ± 0.07	56 ± 1.83	34 ± 0.00	1095.55 ± 7.6
29	798 ± 13.11	45 ± 5.24	58 ± 1.12	14 ± 1.57	32 ± 1.18	101 ± 11.02	36 ± 1.88	1101.33 ± 15.0
30	144.16 ± 6.23	48 ± 5.68	48 ± 5.25	60 ± 2.12	22 ± 0.05	28 ± 5.20	28 ± 0.25	392.72 ± 12.62
31	155 ± 8.3	25 ± 2.62	25 ± 2.36	18 ± 4.5	21 ± 0.07	79 ± 6.23	46 ± 0.69	389.66 ± 17.89
32	472.33 ± 13.5	18 ± 2.0	65 ± 4.25	9 ± 0.12	45 ± 1.08	56 ± 1.02	55 ± 5.68	734.77 ± 12.51
33	534 ± 31	44 ± 1.54	77 ± 3.23	28 ± 2.04	24 ± 1.02	48 ± 0.13	28 ± 0.47	794.33 ± 9.81
34	158 ± 3.46	58 ± 4.03	49 ± 1.40	34 ± 3.50	58 ± 0.04	21 ± 2.32	47 ± 5.12	445.66 ± 17.89
35	353 ± 25.6	27 ± 2.45	58 ± 1.0	25 ± 3.26	21 ± 1.79	55 ± 00	55 ± 0.11	598 ± 3.46
36	798 ± 13.11	49 ± 3.07	55 ± 0.9	45 ± 0.18	40 ± 6.4	48 ± 0.36	41 ± 0.02	1092 ± 13.85
37	171.33 ± 15	78 ± 5.0	24 ± 0.45	11 ± 0.01	14 ± 0.34	75 ± 0.45	25 ± 2.05	399.44 ± 1.0

Numbers 1 to 37 were the sample names (Table 1).

**(a):** 

Number	Amino acids	RT (min)	Molecular weight	1	2	3	4	5	6	7	8	9	10	11	12	13	14	15	16	17
1	Aspartate	1.48	133.10	1.37	1.37	2.03	2.66	5.24	1.34	3.66	1.24	2.47	0.97	3.55	4.05	3.33	0.89	1.06	1.69	1.70
2	Glutamate	2.77	147.13	ND^(a)^	ND^(a)^	ND	ND	ND	ND	ND	ND	ND	ND	ND	ND	ND	ND	ND	ND	ND
3	Asparagine	5.83	132.12	0.25	0.25	ND	0.92	0.39	ND	0.51	4.07	0.32	0.42	0.27	0.17	0.30	0.23	0.28	0.43	0.46
4	Serine	6.03	105.09	1.69	1.69	0.49	1.17	1.14	1.29	1.59	2.02	1.58	1.37	0.92	1.40	0.57	1.61	1.86	2.12	1.18
5	Vitamin U	6.59	199.70	ND	ND	ND	ND	ND	ND	ND	ND	ND	ND	ND	ND	ND	ND	ND	ND	ND
6	Glutamine	6.84	146.15	0.64	0.64	ND	1.89	3.25	0.55	1.46	0.66	ND	0.53	2.12	ND	1.09	ND	0.65	ND	0.98
7	Histidine	7.15	155.15	ND	ND	ND	0.72	ND	0.43	ND	ND	ND	ND	ND	ND	ND	ND	ND	ND	ND
8	Glycine	7.36	75.07	2.53	2.53	0.51	3.51	1.28	1.45	1.77	2.30	1.98	3.05	ND	1.59	1.02	2.04	2.36	2.71	2.28
9	Threonine	7.62	119.12	1.54	1.54	0.45	2.12	0.98	0.78	1.71	1.28	1.24	1.45	1.92	1.48	0.84	1.07	1.08	1.63	2.08
10	Arginine	8.47	174.20	2.81	2.81	1.87	1.87	2.82	4.00	3.04	2.20	5.12	4.70	1.55	2.81	1.59	2.31	2.48	5.12	3.02
11	Alanine	8.97	89.09	ND	ND	ND	11.68	ND	ND	ND	ND	ND	ND	ND	8.12	ND	ND	9.01	16.12	12.94
12	GABA	9.26	103.12	0.38	0.38	0.21	1.54	ND	0.32	0.35	1.26	1.00	0.84	ND	0.72	0.46	0.32	0.34	1.05	1.32
13	Tyrosine	10.43	181.19	2.08	2.08	0.46	3.04	0.69	1.70	1.81	1.43	ND	2.54	ND	1.66	ND	1.24	1.50	2.19	1.28
14	Cystine	12.26	240.30	ND	ND	ND	1.45	ND	ND	ND	ND	1.30	ND	0.45	ND	0.86	ND	ND	ND	0.52
15	Valine	12.56	117.15	2.13	2.13	0.72	5.34	0.76	1.92	2.51	ND	ND	1.07	ND	2.68	ND	1.42	1.20	4.02	3.19
16	Methionine	12.82	149.21	ND	ND	ND	0.80	ND	ND	ND	ND	ND	ND	0.93	ND	ND	ND	0.43	ND	ND
17	Norvaline	13.23	117.15	ND	ND	ND	ND	ND	ND	ND	ND	ND	ND	ND	ND	ND	ND	ND	ND	ND
18	Tryptophan	13.88	204.33	ND	ND	ND	ND	ND	ND	ND	ND	ND	ND	ND	ND	ND	ND	ND	ND	ND
19	Phenylalanine	14.37	165.19	ND	ND	ND	ND	ND	ND	ND	ND	ND	ND	ND	ND	ND	ND	ND	ND	ND
20	Isoleucine	14.60	131.17	1.98	1.98	0.44	4.31	0.69	1.69	2.23	1.13	2.01	0.62	0.39	2.07	0.61	0.89	0.77	2.79	2.00
21	Leucine	15.43	131.17	2.77	2.77	0.54	5.96	1.14	2.68	3.33	1.21	2.63	0.88	0.53	2.87	0.74	1.15	1.40	3.83	3.29
22	Lysine	16.05	146.19	1.68	1.68	0.38	4.04	0.79	0.00	2.44	1.63	1.90	2.21	0.71	1.70	0.72	1.38	1.45	2.95	1.63
Total				**21.86 **	**21.86 **	**8.09 **	**53.04 **	**19.18 **	**18.16 **	**26.41 **	**20.44 **	**21.56 **	**20.65 **	**13.34 **	**31.32 **	**12.13 **	**14.55 **	**25.87 **	**46.66 **	**37.88 **

**(b):** 

Number	Amino acids	RT (min)	Molecular weight	18	19	20	21	22	23	24	25	26	27	28	29	30	31	32	33	34	35	36	37
1	Aspartate	1.48	133.10	2.76	3.21	1.44	2.88	1.45	0.71	2.75	0.46	1.10	1.87	1.98	1.56	0.56	1.21	1.16	1.62	1.28	0.49	1.94	1.02
2	Glutamate	2.77	147.13	ND	ND	ND	ND	ND	ND	ND	ND	ND	ND	ND	ND	ND	ND	ND	ND	ND	ND	ND	ND
3	Asparagine	5.83	132.12	0.89	0.96	0.42	0.97	ND	ND	0.22	0.39	0.21	ND	ND	0.32	ND	0.35	ND	0.24	1.26	ND	0.52	0.37
4	Serine	6.03	105.09	1.12	1.91	0.92	1.04	2.17	1.11	0.56	0.69	1.14	1.12	1.96	1.01	0.62	1.02	1.01	1.11	3.17	0.79	1.74	1.15
5	Vitamin U	6.59	199.70	ND	ND	ND	ND	ND	ND	ND	ND	ND	ND	ND	ND	ND	ND	ND	ND	ND	ND	ND	ND
6	Glutamine	6.84	146.15	1.31	1.52	0.98	1.87	ND	ND	1.07	ND	ND	ND	ND	0.53	ND	ND	0.57	ND	ND	ND	ND	ND
7	Histidine	7.15	155.15	0.48	0.56	ND	0.91	ND	ND	ND	ND	ND	ND	ND	ND	ND	0.30	ND	ND	0.53	ND	0.39	0.31
8	Glycine	7.36	75.07	2.65	3.13	1.96	3.95	1.75	1.69	1.08	2.04	2.27	2.19	3.37	1.41	1.46	2.24	2.31	1.51	6.33	1.47	5.37	2.03
9	Threonine	7.62	119.12	2.83	3.63	1.84	2.34	1.30	0.72	0.73	0.58	0.94	1.57	1.61	1.09	0.69	2.10	1.71	1.04	1.74	0.70	1.34	1.19
10	Arginine	8.47	174.20	2.52	3.12	1.98	2.35	5.37	2.47	1.74	1.64	2.19	2.66	3.58	2.66	1.34	2.80	3.01	2.41	8.46	1.88	2.63	2.91
11	Alanine	8.97	89.09	9.27	13.57	8.60	12.80	11.68	9.04	ND	8.03	9.26	9.84	12.89	4.36	7.60	15.30	11.57	6.51	23.85	6.03	16.34	6.24
12	GABA	9.26	103.12	1.26	1.83	0.99	1.35	0.48	1.79	0.20	1.74	1.13	1.63	4.36	0.44	6.72	1.16	0.57	0.44	0.77	0.23	2.35	0.97
13	Tyrosine	10.43	181.19	2.33	1.36	1.21	3.30	2.22	1.44	0.64	1.51	1.84	1.93	3.26	1.36	1.09	1.17	1.71	1.63	4.45	1.13	2.20	1.82
14	Cystine	12.26	240.30	1.30	0.80	0.65	1.64	0.38	ND	ND	ND	ND	ND	ND	ND	ND	0.76	ND	ND	0.57	ND	0.78	ND
15	Valine	12.56	117.15	4.34	5.38	2.43	6.05	2.97	1.49	0.70	1.29	1.69	2.65	3.05	1.19	1.62	3.60	3.42	1.67	16.12	ND	4.41	1.44
16	Methionine	12.82	149.21	0.69	0.35	ND	0.98	ND	ND	ND	ND	ND	ND	ND	ND	ND	ND	ND	ND	ND	ND	ND	ND
17	Norvaline	13.23	117.15	ND	ND	ND	ND	ND	ND	ND	ND	ND	ND	ND	ND	ND	ND	ND	ND	ND	ND	ND	ND
18	Tryptophan	13.88	204.33	ND	ND	ND	ND	ND	ND	ND	ND	ND	ND	ND	ND	ND	0.43	ND	ND	0.64	ND	0.69	ND
19	Phenylalanine	14.37	165.19	2.76	2.53	1.13	3.36	1.42	0.83	ND	0.42	0.46	0.38	0.98	0.48	0.62	1.65	0.87	0.59	2.93	0.62	1.73	0.74
20	Isoleucine	14.60	131.17	2.95	3.43	1.29	3.98	2.07	0.95	0.44	0.92	1.05	1.50	1.70	0.85	1.06	1.92	2.67	1.11	ND	0.68	ND	ND
21	Leucine	15.43	131.17	4.74	5.82	2.41	6.59	3.99	1.81	0.71	1.03	1.21	1.47	1.95	1.24	1.61	3.32	3.35	1.62	7.59	0.96	3.14	1.23
22	Lysine	16.05	146.19	2.88	3.01	1.08	4.47	2.48	1.63	0.65	1.62	1.61	1.21	2.70	1.16	0.78	1.68	1.40	1.36	6.27	1.08	2.74	1.59
Total				**47.05**	**56.14**	**29.35**	**60.83**	**39.74**	**25.68**	**11.49**	**22.34**	**26.10**	**30.01**	**43.38**	**19.65**	**25.77**	**41.00**	**35.33**	**22.85**	**85.97**	**16.08**	**48.31**	**23.01**

Numbers 1 to 37 were the sample names (Table 1). ^(a)^ND: not detected.
